# 546 Health Disparities Among Rural Burn Patients

**DOI:** 10.1093/jbcr/irac012.174

**Published:** 2022-03-23

**Authors:** Robert P Duggan, Kimberley C Brondeel, William L Mahony, Grant Torres, Alen Palackic, Ludwik K Branski

**Affiliations:** University of Texas Medical Branch, Galveston, Texas; University of Texas Medical Branch, Galveston, Texas; The University of Texas Medical Branch, Galveston, Texas; University of Texas Medical Branch, Galveston, Texas; University of Texas Medical Branch, Galveston, Texas; University of Texas Medical Branch, Galveston, Texas

## Abstract

**Introduction:**

Socioeconomic status is a risk factor for sustaining a burn and for burn mortality. Patients from rural areas make up a minority of the population but are frequently more isolated from life-saving care and burn centers. Lower socioeconomic status patients may delay seeking treatment of their burns for concern over medical costs, time away from work, and overall distance from accredited burn centers. We aim to explore disparities in burn outcomes at our institution based on patient socioeconomic status.

**Methods:**

Between January 2020 and January 2021, patients presenting for management of acute burns were reviewed. Patient demographics and outcomes were collected, including time to presentation, total body surface area burned, presence of inhalational injury, and mortality. Patient socioeconomic status and rural designations were assigned based on a validated metric derived from Census endpoints, with higher scores reflecting lower socioeconomic status.

**Results:**

A total of 524 patients were identified. Overall, 30% of our patients were from areas defined as being small towns or rural by the Census. Racial demographics did not differ between rural and urban areas (p = 0.099), but Hispanic ethnicity was less common (16% vs. 29%, p = 0.002). Rates of alcohol, tobacco, and illicit drug use did not differ between groups. Compared to the urban/suburban cohort, rural patients were from less affluent areas (63.6 vs. 58.5, p = 0.001) and traveled farther to our center (112 miles vs. 70 miles, p = 0.029). Despite these distances, rural patients did not have a higher rate of delayed presentation (35.7% vs. 43.3%, p = 0.105), or longer average time to presentation (3.4 days vs 4.4 days, p = 0.222). Flame burns were the most common mechanism overall (44.3%) and were significantly more common in the rural population (59.2% vs. 37.8%, p < 0.001), Scalds, the second most common burn mechanism (25.9%), occurred less frequently in rural patients (18.5% vs. 29.2%, p = 0.011). Controlling for age, TBSA, inhalational injury, and ventilator requirement, patients from rural areas were at a significantly higher risk of mortality (OR 24, p = 0.024).

**Conclusions:**

Rural burn patients face many challenges receiving appropriate care following a burn. They frequently come from less affluent backgrounds, limiting their ability to access care, and they must travel greater distances to a qualified burn surgeon. Despite these barriers, our rural patient population did not present any later following a burn compared to our more urban patients. Rural patients sustained more extensive burns but were not hospitalized at a greater rate. Even when controlling for numerous factors associated with burn mortality, rural patients were still at an increased risk. Burn prevention strategies targeting rural communities should address the unique challenges facing these areas.

